# Extramedullary Plasmacytoma of the Oral Cavity in a Young Man: a Case Report

**Published:** 2016-06

**Authors:** Narges Gholizadeh, Masoumeh Mehdipour, Bita Rohani, Vahid Esmaeili

**Affiliations:** 1 Dept. of Oral Medicine, School of Dentistry, Tehran University of Medical Sciences, Tehran, Iran.; 2 Dept. of Oral Medicine, School of Dentistry, Shahid Beheshti University of Medical Sciences, Tehran, Iran.; 3 Dept. of Oral Medicine, School of Dentistry, AJA University of Medical Sciences, Tehran, Iran.; 4 Dept. of Oral and Maxillofacial Pathology, School of Dentistry, AJA University of Medical Sciences, Tehran, Iran.

**Keywords:** Extramedullary Plasmacytoma, Multiple Myeloma, Oral Cavity

## Abstract

Extramedullary plasmacytomas are rare solitary soft tissue tumors that arise from proliferations of malignant transformed monoclonal plasma cells and can be diagnosed through biopsy and histopathologic examination. These lesions are closely associated with multiple myelomas, which should be ruled out in all these cases by necessary laboratory and radiographic examinations. A 25-year-old man was referred to our clinic with a rapidly-growing painless lesion measuring about 2.5×3×3 cm in the palatal side of the left maxillary second and third molar teeth. A diagnosis of solitary plasmacytoma was made on the basis of clinical, radiographic, and histopathological findings. Early diagnosis of extramedullary plasmacytomas is of great importance. Radiotherapy is the common modality of treatment with or without adjuvant chemotherapy. Progression to multiple myeloma is possible; thus, close follow-up of the patient is essential after completion of the therapeutic procedure.

## Introduction

Plasma cell tumors consist of extra medullary plasmacytoma (EMP), multiple myeloma (MM) and solitary bone plasmacytoma (SBP). Plasmacytoma is a plasma cell neoplasm of bone marrow that is in the end stage of B lymphocyte maturation.[1] It is a solitary tumor of neoplastic monoclonal plasma cells proliferation in either bone or soft tissue that is known as EMP.[2] Solitary plasmacytoma may be an isolated tumor in any region of body or the first manifestation of a subsequent MM. EMP comprises up to 3% of all plasma cell tumors. These tumors are more common in men, particularly in their sixth to eighth decades of life.[3] Extramedullary lesions may occur in the absence of bone involvement, especially in the head and neck region.[4] Approximately 90% of EMPs are found in the head and neck region. They commonly involve the nasal cavity, paranasal sinuses, tonsillar fossa, and oral cavity.[4] Yoshimura *et al.* reported 2 cases of plasmacytoma in the oral region.[5]

The etiology of this disease is still unknown, but viruses, overdose irradiation, chronic stimulation, and gene disorders in the reticuloendothelial system have been suggested as the probable etiologic factors.[6]

Some authors consider it to be unrelated to MM, even though both of them are similar in microscopic view.[7] Currently, the accepted criteria obtained by using MRI, flow cytometry, and polymerase chain reaction (PCR) include the solitary extramedullary mass of clonal plasma cells, bone marrow plasma cell infiltration (≤5.0% of all nucleated cells), absence of osteolytic lesions or other tissues involvement (no evidence of myeloma elsewhere), and absence or low level of serum or urinary monoclonal immunoglobulin.[4, 8]

Plasmacytomas can be graded as lesions of minimal to severe dysplasia.[1] In this article, we reported an EMP in an otherwise healthy young man.

## Case Report

**Figure 1:**
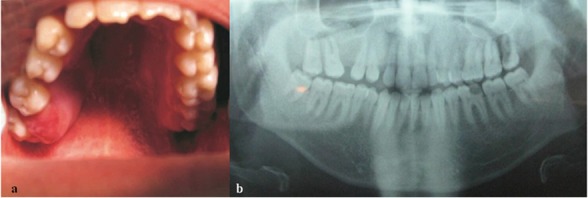
a: The photograph showing the tumor in the palatal side of left maxillary second and third molars b: Normal view in panoramic radiograph

A 25-year-old man was referred to the Department of Oral Medicine with complaint of a swollen lesion in the oral cavity. The patient reported a rapidly-growing painless lesion during the last 20 days. On physical examination, an erythematous and ulcerative tumor measured about 2.5´3´3 cm was observed in the palatal side of left maxillary second and third molar teeth (Figure 1a).

The clinical features including ulceration, mobility of the adjacent teeth, and rapid growth of the lesion suggested some differential diagnosis such as minor salivary gland tumors, osteosarcoma, lymphoma, and aggressive reactive lesions. The teeth adjacent to the lesion were mobile, without decay, slightly sensitive to percussion, and vital in vitality test. For further evaluation, panoramic and periapical radiographs were requested (Figure 1b).

Laboratory test results were negative for any evidence of anemia, thrombocytopenia, and hypercalcemia. 

An incisional biopsy of 1×1×1 cm was performed under local anesthesia. The mass of the lesion was friable and strongly hemorrhagic during biopsy. To control bleeding, various methods were used including local pack with gas, using hydrogen peroxide, and suturing the region. Yet, these methods were not efficient and, finally, the greater palatine artery was ligated with a deep suture. 

**Figure 2:**
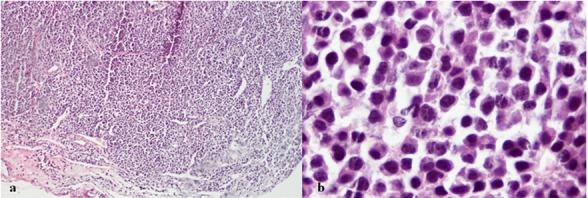
Histopathologic views of the lesion; a: with 100x magnification, b: with 400x magnification

The microscopic evaluation showed heavy infiltration of plasma cells arranged in large sheets or nodules. Microscopically, the plasma cells showed varying degrees of differentiation with sparse stroma. The majority of plasma cells were poorly or moderately differentiated with large and distinct nuclei, but the minorities were well differentiated. Many mitosis and occasional debris containing macrophages were seen. There were modest number of lymphocytes in fibrosis septa between the lobules of plasma cells and focal surface ulceration (Figure 2a and 2b). Bone marrow biopsy failed to show evidence of myeloma. The skeletal survey was also unremarkable. Serum proteins and immunoglobulin levels were within normal limits. Urine examination showed no Bence Jones protein.

**Figure 3:**
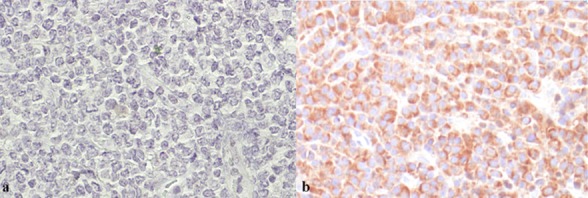
Immunohistochemical staining for kappa, a: lambda b: demonstrating monoclonality of plasma cells (Magnification 200x)

A diagnosis of solitary plasmacytoma was made on the basis of clinical, radiographic, and histopathological findings. Immunohistochemical staining was used to confirm the monoclonality of plasma cells (Figure 3a and 3b). The patient received radiotherapy; 40 Gy was fractioned in 4 weeks. After a while, all clinical symptoms of the lesion disappeared and he was in the same condition up to one year. Thereafter, the patient was not accessible for follow-up.

## Discussion

In addition to the division mentioned at the beginning of the article, plasmacytoma traditionally is divided into medullary and extramedullary types, which can be either solitary or multiple in distribution. The most common type of plasma cell tumors is the generalized medullary form (multiple myeloma).[4] EMP makes up approximately 3% of all plasma cell tumors.[3] In EMPs, pain is commonly absent, unless there is some secondary infection or bone destruction.[9] In this case, the patient reported painless growth of the lesion. EMP occurs mainly in the fifth and sixth decades of life with a higher prevalence in men.[10] The incidence is higher among individuals of advanced age, with a mean age at diagnosis of 64 years. More than 95% of cases occur in individuals older than 40 years;[10] while, we reported this lesion in a patient younger than 30 years old.

Nearly 80 percent of extramedullary plasmacytomas originate from the upper respiratory tract (with nose and paranasal sinuses as the most common places), followed by nasopharynx, tonsils and oropharynx.[11-12]Rawat *et al.* reported an extramedullary plasmacytoma on one side of the nasal cavity that completely occluded the nasal cavity on the same side and the septum was pushed to the opposite side.[12] While in our patient, the lesion occurred in the maxilla which is a rare location for the occurrence of this lesion. One patient was reported by Singh *et al.* with complaints of swelling in the maxillary region. Radiographic evaluation showed a dense opacity overlying the maxillary sinus with destruction of its floor and lateral walls.[13]

We reported this case because of the low age of our patient (opposed to the majority of reported cases), rare occurrence of the lesion in the maxilla, and also the rarity of extramedullary type of plasmacytoma compared with its bony type.

Plasmacytoma is clinically similar to chronic inflammatory diseases, plasma cell gingivitis, and lymphomas that are distinguishable histopathologically. In fact, microscopic view of the lesion is the only diagnostic tool for differentiating plasmacytoma from other lesions. Monoclonal proliferation is suggestive of neoplasm superimposed to inflammatory lesions. Radiographically, EMP can erode the bone and make it difficult to be distinguished from a bony lesion.[14] Solitary plasmacytoma is different from MM due to lack of plasma cell infiltration in a bone marrow biopsy.[3]

Histopathologic features of EMP show a connective tissue greatly infiltrated by plasma cells which are like focal sheets, small islands, or plasmacytoid nodules.[5] Moreover, in this report, majority of plasma cells were poorly or moderately differentiated with large and distinct nuclei, and the minorities were well-differentiated. Since the granulomas associated with dental infection demonstrate a large number of plasma cells, special attention should be considered when diagnosing a lesion as solitary myeloma. Because of the common dense infiltration of plasma cells associated with inflammatory lesions of the oral tissues, the diagnosis of plasmacytoma and plasma cell granuloma is difficult by using only histopathologic findings. Thus, flow cytometry is recommended in such situations.[15]

The rate of progression of EMP to MM is 15-20%. Harwood *et al.* reported the high rate of conversion to MM if EMP involved the underlying bone.[16] In some cases that were treated with radiation, 0.0-11% local recurrence was reported. In this situation, the recurrence in bone is concomitant with progression rate of multiple myeloma.[7]

EMP is a rare lesion that comprises about 3% of all plasma cell neoplasms. So far, the majority of lesions have been seen in men of older ages;[3] while, our patient was a young man, which is a very rare case. Barros *et al.* (2011) reported[10] a case of oral EMP in a 70-year-old man with a long history of smoking and alcohol drinking; while, our case was a 25-year-old man with no history of smoking and alcohol consumption.

Management of EMP primarily involves local eradication of the lesion. EMPs are highly-radiosensitive tumors.[17] Up to now, there is no established radiotherapy protocol for treatment of plasmacytoma. However, most studies accepted that 46 Gy is the best in local control with minimal toxicity.[18] Hence, our patient was treated by oncologists with approximately the same dose (40 Gy). For esthetic reasons, radical surgery should be avoided in management of solitary lesions in the head and neck region. When accessible, surgical removal may be considered for treatment of EMPs in other areas. If the surgical margins are also involved, the patient should receive adjuvant radiotherapy.[19]
